# Incidental Adrenal Hemangioma: A Diagnostic and Management Challenge

**DOI:** 10.1210/jcemcr/luaf209

**Published:** 2025-09-18

**Authors:** Abhinay Jain, Alka Rana, Phibakordor L Nonglait, Pragya Mangla, Nishant Raizada, Sri Venkata Madhu

**Affiliations:** Department of Endocrinology, University College of Medical Sciences and GTB Hospital, Delhi 110095, India; Department of Pathology, Medanta-The Medicity Hospital, Gurugram 122001, India; Department of Medicine, Civil Hospital, Shillong 793004, India; Department of Endocrinology, University College of Medical Sciences and GTB Hospital, Delhi 110095, India; Department of Endocrinology, University College of Medical Sciences and GTB Hospital, Delhi 110095, India; Department of Endocrinology, University College of Medical Sciences and GTB Hospital, Delhi 110095, India

**Keywords:** adrenal, hemangioma, tumor, adrenalectomy

## Abstract

Adrenal hemangioma is a rare, benign vascular tumor often discovered incidentally during imaging. Here, we present the case of a 43-year-old male individual who was incidentally found to have a right adrenal mass during evaluation for polycythemia. Initial imaging revealed a 3.1 × 2.3 cm mass. The unenhanced computed tomography (CT) attenuation was 31 Hounsfield units (HU), and the post-contrast attenuation was approximately 61 HU. Hormonal evaluations, including cortisol, dehydroepiandrosterone sulfate (DHEA-S), aldosterone, renin, and metanephrines, were all within normal limits. The patient was managed conservatively, with close follow-up. Over the course of 2 years, the lesion remained stable in size until a sudden increase was noted, reaching 4.3 × 3.3 cm, with magnetic resonance imaging (MRI) revealing necrotic areas and peripheral enhancement. Due to the lesion's rapid growth and indeterminate features, the patient underwent a laparoscopic right adrenalectomy. The histopathological analysis confirmed the diagnosis of a cavernous adrenal hemangioma. This case emphasizes the importance of recognizing adrenal hemangiomas, as they can mimic more aggressive adrenal tumors and require careful monitoring.

## Introduction

Adrenal hemangiomas are rare, benign vascular tumors of endothelial cells typically found incidentally during imaging for unrelated conditions, as they are usually asymptomatic [1, 2]. Their prevalence is estimated at 0.56% in radiological studies and about 1 per 10 000 in autopsy series [2, 3]. Despite their benign nature, the differential diagnosis can be challenging, especially given their potential resemblance to more concerning adrenal pathologies such as pheochromocytoma or adrenal cortical carcinoma [2, 4, 5]. Understanding the unique features of adrenal hemangioma, both in imaging and histopathology, is essential for proper diagnosis and management. Here, we present a case of an adrenal hemangioma discovered incidentally during the evaluation of a patient with polycythemia. The case involved a comprehensive biochemical and imaging workup alongside long-term follow-up, offering valuable insights into the natural history and diagnostic characteristics of this rare tumor.

## Case Presentation

The 43-year-old male patient was detected to have polycythemia during routine screening prior to blood donation. His hemoglobin was found to be 18.3 g/dL (183 g/L) (reference range: 13.2-16.5 g/dL; 132-165 g/L), and his hematocrit was 54% (reference range, 41%-49%). He was asymptomatic at the time of presentation, with no complaints of aquagenic pruritus, headaches, visual disturbances, or hyperviscosity-related symptoms. The patient was a nonsmoker. There was a history of a single episode of hypertension 2 years back, when his blood pressure was recorded at 140/110 mmHg. He had a history of COVID pneumonia 1.5 years back.

## Diagnostic Assessment

**Figure 1.**
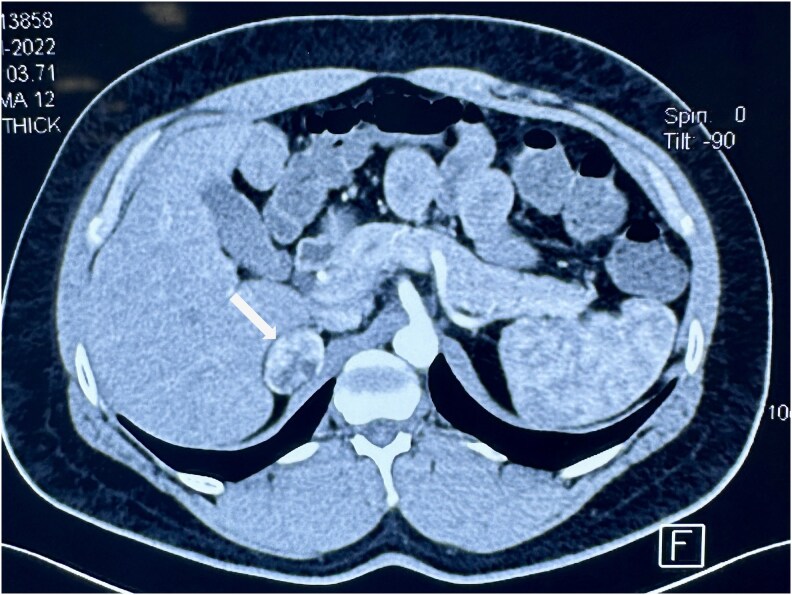
The axial image of the contrast-enhanced computed tomography scan (venous phase) showing a well-defined round to oval-shaped heterogeneous enhancing mass lesion measuring 3.1 × 2.3 cm (white arrow) arising from the right adrenal gland.

Biochemical and genetic workup for polycythemia was negative (Table 1). On further evaluation, a contrast-enhanced computed tomography (CECT) scan of the abdomen was performed to investigate any underlying cause of secondary polycythemia. This imaging revealed a right adrenal mass measuring 3.1 × 2.3 cm with heterogeneous enhancement. The unenhanced CT attenuation was 31 Hounsfield units (HU), and the post-contrast attenuation was approximately 61 HU (Fig. 1). The lesion appeared well-defined, originating from the medial limb of the right adrenal gland. No significant abnormalities were noted in the left adrenal gland. Therefore, the patient was referred to the endocrine department for further evaluation.

**Table 1.** Baseline laboratory investigations of the patient for polycythemia and adrenal mass evaluation

Investigation	Result	Reference range
**Hemoglobin**	18.3 g/dL (183 g/L)	13.2-16.5 g/dL (132-165 g/L)
**Hematocrit**	54%	41-49%
**Total leukocyte count**	10 100/µL (10.1 × 10⁹/L)	4000–11 000/µL (4.0-11.0 × 10⁹/L)
**Platelet count**	364 × 10³/µL (364 × 10⁹/L)	150-450 × 10³/µL (150-450 × 10⁹/L)
**Erythrocyte sedimentation rate**	2 mm/h (2 mm/h)	0-15 mm/h (0-15 mm/h)
**Urea**	20 mg/dL (3.3 mmol/L)	7-20 mg/dL (2.5-7.1 mmol/L)
**Creatinine**	0.9 mg/dL (79.56 µmol/L)	0.74-1.35 mg/dL (65.4-119.3 µmol/L)
**Sodium**	140 mEq/L (140 mmol/L)	135-145 mEq/L (135-145 mmol/L)
**Potassium**	4.75 mEq/L (4.75 mmol/L)	3.5-5.1 mEq/L (3.5-5.1 mmol/L)
**Calcium**	9.92 mg/dL (2.48 mmol/L)	8.5-10.5 mg/dL (2.12-2.62 mmol/L)
**Phosphorus**	2.80 mg/dL (0.90 mmol/L)	2.5-4.5 mg/dL (0.81-1.45 mmol/L)
**D-Dimer**	0.15 mg/L FEU (0.82 nmol/L FEU)	<0.5 mg/L FEU (2.74 nmol/L FEU)
**Interleukin-6 (IL-6)**	1.5 pg/mL (1.5 pg/mL)	<7 pg/mL (<7 pg/mL)
**Erythropoietin**	15.5 IU/L	4.1-19.9 IU/L
**Ferritin**	87.26 ng/mL (196.07 pmol/L)	30-400 ng/mL (67.41-898.8 pmol/L)
***JAK 2* (V617 F) pathogenic variant**	Not detected	Negative
***MPL* mutation**	Not detected	Negative
***CALR* mutation**	Not detected	Negative
**Serum dehydroepiandrosterone sulfate (DHEA-S)**	295.20 µg/dL (7.9 µmol/L)	88.9-427 µg/dL (2.40-11.53 µmol/L)
**Serum aldosterone**	13.5 ng/dL (374.5 pmol/L)	2.52-39.2 ng/dL (81-1087.40 pmol/L)
**Direct renin concentration**	23.67 mIU/L (15.07 ng/L)	4.40-46.10 mIU/L (2.80-29.36 ng/L)
**Aldosterone-to-renin ratio (ARR)**	0.57 ng/dL per mIU/L (24.85 pmol/L per ng/L)	<2.4 ng/dL per mIU/L (<144 pmol/L per ng/L)
**24-hour urine metanephrine**	30.66 µg/24 hours (155.44 nmol/24 hours)	<350 µg/24 hours (<1774.5 nmol/24 hours)
**24-hour urine normetanephrine**	353.5 µg/24 hours (1930.1 nmol/24 hours)	<600 µg/24 hours (<3276 nmol/24 hours)

Abbreviations: CALR, calreticulin; JAK2, Janus kinase 2; MPL, myeloproliferative leukemia virus.

**Figure 2.**
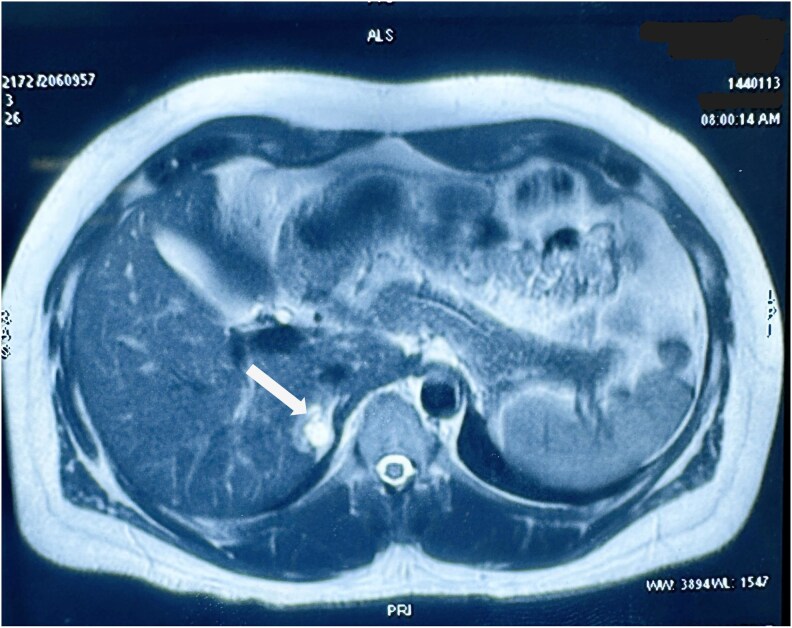
Axial T2-weighted magnetic resonance imaging showing a heterogeneously hyperintense lesion (white arrow) in the right adrenal region, consistent with cystic or vascular components. The lesion measures approximately 3.3 × 2.2 × 3.1 cm.

A detailed hormonal workup was conducted to assess for adrenal overactivity. The overnight dexamethasone suppression test (ONDST) returned a cortisol level of 0.9 µg/dL (24.82 nmol/L) (normal <1.8 µg/dL; < 49.65 nmol/L). The rest of the biochemical workup for adrenal hormone excess was within normal limits (Table 1). Magnetic resonance imaging (MRI) was performed to further characterize the adrenal lesion. The MRI revealed a well-defined right adrenal mass measuring 3.3 × 2.2 × 3.1 cm (AP x TR x CC) with heterogeneous signal intensity. The lesion appeared mildly hyperintense on T2-weighted images and iso-hypointense on T1-weighted images. The absence of signal drop on out-of-phase imaging suggested a lipid-poor lesion, and areas of cystic degeneration were noted (Fig. 2). Given the patient's history of hypertension and specific imaging findings, the possibility of pheochromocytoma was considered, despite normal metanephrine levels. Further evaluation was planned to investigate this possibility. Considering the presence of polycythemia alongside an adrenal mass, Pacak-Zhuang syndrome—associated with *HIF2A* mutations and characterized by pheochromocytoma or paraganglioma, polycythemia, and somatostatinoma—was also considered. An eye examination was conducted to assess for ocular abnormalities frequently associated with this syndrome [6], and the results were normal. Gallium 68-DOTANOC positron emission tomography (PET) (DOTANOC is a conjugate of gallium-68-labeled 1,4,7,10-tetraazacyclododecane-N,N′,N′′,N′′′-tetraacetic acid [DOTA] and the somatostatin analog 1-Nal3-octreotide [NOC]) scan revealed a well-defined, non-DOTANOC-avid, arterially enhancing heterogenous lesion in the right adrenal gland with areas of hypodensity, while the rest of the scan was unremarkable. The radiologic impression at the time was a nonfunctional right adrenal mass with indeterminate characteristics, with possibilities including a lipid-poor adenoma or other lipid-poor masses.

**Figure 3.**
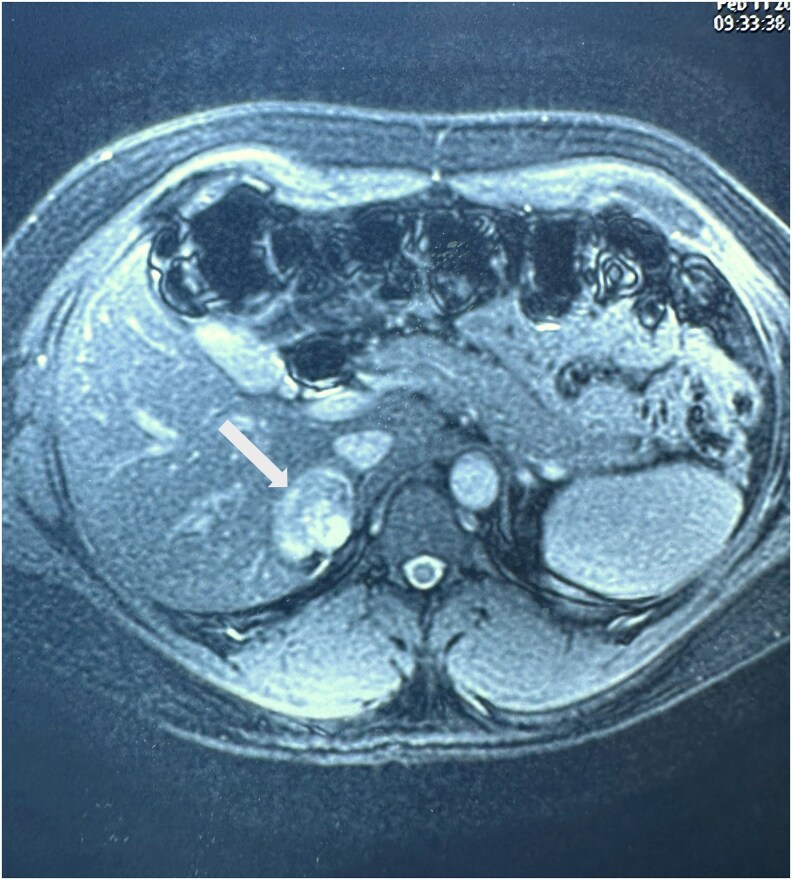
Follow-up axial T2-weighted magnetic resonance imaging of the abdomen showing a well-defined, oval-shaped, heterogeneously hyperintense lesion (white arrow) in the right adrenal gland, measuring 4.3 × 3.3 cm transversely and 3.7 cm craniocaudally. The lesion shows interval growth with multiple non-enhancing necrotic areas.

The patient was asymptomatic and was managed conservatively with close follow-up. A follow-up CECT scan conducted 6 months later did not reveal any change in the size of the lesion (Table 2). Repeat 24-hour urine metanephrine was 42.06 µg/24 hours (215.98 nmol/24 hours) (normal <350 µg/24 hours; < 1774.5 nmol/24 hours) and 24-hour urine normetanephrine was 177 µg/24 hours (966.42 nmol/24 hours) (normal <600 µg/24 hours; < 3276 nmol/24 hours). His polycythemia also resolved after 3 phlebotomy sessions with a hemoglobin of 15.5 g/dL (155 g/L). The patient underwent serial ultrasound evaluations every 6 months to monitor the right adrenal mass (Table 2). The size of the lesion remained relatively stable, but at 2 years of follow-up, the lesion showed significant growth. An MRI scan of the abdomen was done, confirming an increase in lesion size (Table 2, Fig. 3). Due to the sudden increase in the size of the lesion, the possibility of adrenocortical carcinoma was considered. Therefore, an 18-fluorodeoxyglucose positron emission tomography (18-FDG PET) scan was performed, which revealed a mildly FDG-avid nodular soft tissue lesion in the right adrenal gland (SUV max: 5.8). No other significant FDG-avid lesions were noted.

**Table 2.** Serial imaging and follow-up of right adrenal mass

CECT scan (6 months from baseline)	Right adrenal mass measuring 3.3 × 3.1 × 2.4 cm, with multiple areas of non-enhancing hypodensity suggestive of necrosis. Absolute washout: 51% and relative washout: 43%, with no evidence of calcifications.
Ultrasound abdomen (6 months from baseline)	Well-defined hypoechoic nodular lesion seen in relation to right suprarenal gland. It measures 32 × 32 × 23 mm with a volume of 12.7 cc.
Ultrasound abdomen (12 months from baseline)	Size of the lesion: 30 × 25 × 22 mm with a volume of 9.1 cc.
Ultrasound abdomen (18 months from baseline)	Size of the lesion: 27 × 26 × 25 mm with a volume of 9.5 cc.
Ultrasound abdomen (24 months from baseline)	Size of the lesion: 39 × 37 × 30 mm with a volume of 23 cc.
MRI abdomen (24 months from baseline)	A well-defined oval-shaped altered signal intensity mass in the right suprarenal region measuring 4.3 × 3.3 cm transversely and 3.7 cm in craniocaudal dimension; predominantly T1 hypointense and T2 hyperintense signals; areas of necrosis within the lesion; post-contrast images show peripheral enhancement.

Abbreviations: CECT, contrast-enhanced computed tomography; MRI, magnetic resonance imaging.

## Treatment

Given the progressive enlargement of the mass, a decision was made to proceed with surgical excision. Preoperative evaluation to rule out catecholamine excess was conducted, which revealed plasma free metanephrine: 39.3 pg/mL (199.25 pmol/L) (reference range, 7.9-88.7 pg/mL; 40.1-449.7 pmol/L), and plasma free normetanephrine: 65.2 pg/mL (355.9 pmol/L) (reference range, 20.1-135.4 pg/mL; 109.7-739.3 pmol/L). Subsequently, the patient underwent laparoscopic right adrenalectomy.

## Outcome and Follow-up

**Figure 4.**
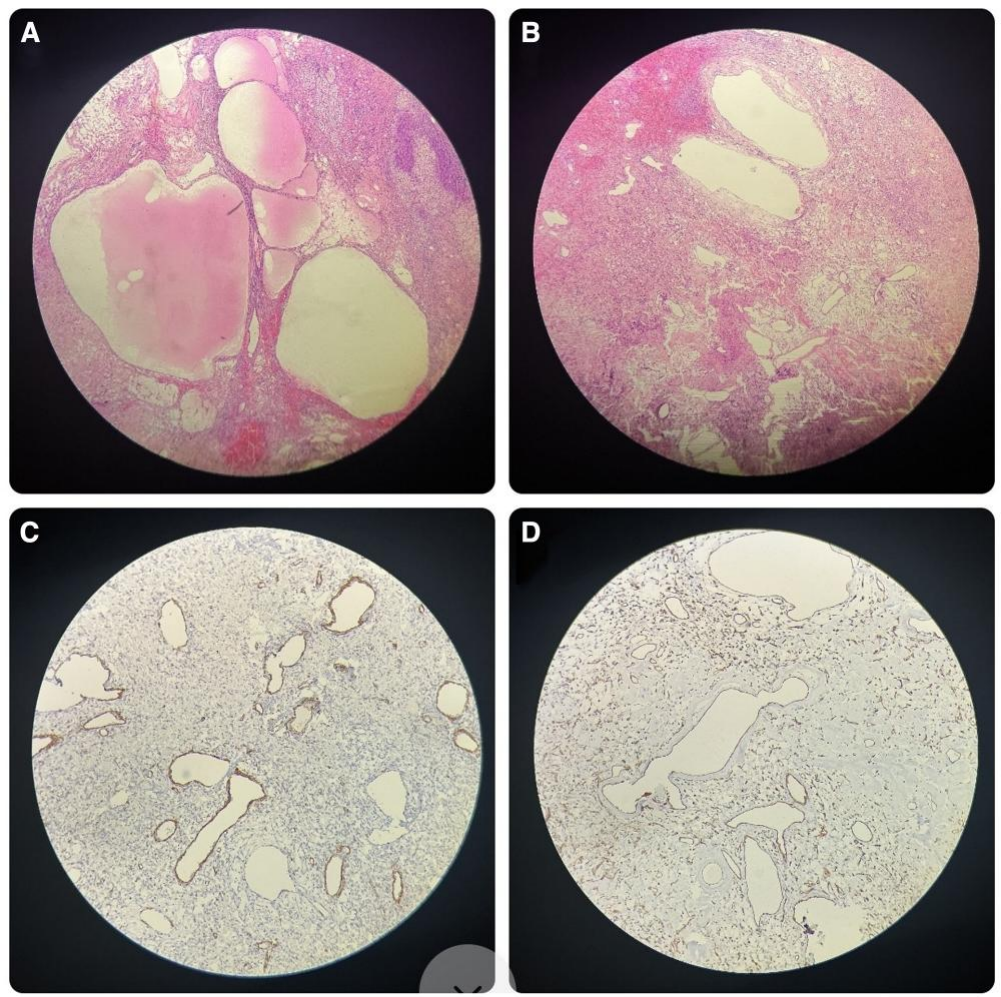
Adrenal cavernous hemangioma. Low-power view (Fig. 4A, H&E, 4x) shows large, thin-walled, blood-filled vascular spaces separated by fibrous septa within the adrenal cortex. Medium-power view (Fig. 4B and H&E, 10x) highlights cavernous vascular spaces lined by flattened endothelial cells with intervening fibrous stroma. CD31 immunohistochemistry (Fig. 4C, 10x) shows strong membranous positivity in the endothelial lining of vascular spaces. Smooth muscle actin (SMA) immunostaining (Fig. 4D, 10x) highlights smooth muscle actin-positive pericytes and vessel walls around the dilated vascular spaces.

The surgery was uneventful, and the adrenal mass was completely excised. Histopathological examination showed a well-circumscribed mass, composed of numerous blood vessels of varying calibers, surrounded by collagen and mature adipocytes, with areas of hemorrhage and necrosis. The immunohistochemical analysis demonstrated positive staining for smooth muscle actin (SMA) and CD31, while staining for S-100 was negative. (Figure 4). These features were consistent with the diagnosis of adrenal cavernous hemangioma. The patient had an uneventful postoperative recovery, and follow-up imaging over 1 year demonstrated no recurrence of the adrenal mass.

## Discussion

Adrenal hemangiomas are rare tumors, with fewer than 200 cases reported in the literature to date [1, 2]. The cavernous subtype is more frequently observed, while the capillary subtype remains rare [7, 8]. They are often discovered incidentally, as in our case, where the adrenal mass was detected during the evaluation for polycythemia. A case series involving 40 patients found that 70% of the cases were asymptomatic [2]. The increasing detection of adrenal hemangiomas can be attributed to the widespread use of advanced imaging modalities such as CT and MRI scans. These tumors are most commonly found in middle-aged individuals, with a slight female predominance reported in some studies, although this case involves a middle-aged male [2, 8].

In terms of imaging, adrenal hemangiomas exhibit distinct characteristics that can aid in diagnosis. On ultrasound, they lack specific defining features, generally appearing as masses with variable size and echotexture [2, 9]. In our case, the lesion appeared hypoechoic on ultrasound. On CT, they appear as well-defined masses, often with areas of necrosis and nodular peripheral enhancement, with or without centripetal filling on delayed phases. They usually have unenhanced CT attenuation values greater than 10 HU [2, 9-11]. Calcifications, which are more common in larger lesions, may also be present. Calcifications can result from phleboliths or dystrophic changes following previous hemorrhages. In a review of 101 cases, calcifications were reported in 43% of the cases of adrenal hemangioma [1]. However, no calcifications were observed in our patient. On MRI, they are usually hypointense on T1 with a remarkable hyperintensity on T2-weighted images (lightbulb sign). Also, peripheral spotty and centripetal enhancement is typically seen on dynamic MRI studies [9, 11]. In our case, the adrenal hemangioma exhibited typical MRI features. Additionally, FDG PET scans usually show low metabolic activity in such lesions [5, 12], which was consistent with our case. Hormonal evaluation in these patients is typically normal, as adrenal hemangiomas are generally hormonally silent and non-secretory [4, 12]. A comprehensive hormonal evaluation was done in the index case also, which was normal.

Differentiating between benign adrenal hemangioma and more aggressive lesions, such as adrenal cortical carcinoma or pheochromocytoma, can be difficult preoperatively [4, 8, 12]. This is because adrenal hemangioma often displays unenhanced CT attenuation values greater than 10 HU, and may exhibit calcifications and areas of necrosis. Moreover, these tumors are typically large and can progressively increase in size over time. Misdiagnosis can lead to unnecessary interventions or inappropriate preoperative management, particularly with regard to pheochromocytoma, which requires specific medical preparation. In our patient, normal metanephrine and normetanephrine levels helped rule out pheochromocytoma as a differential diagnosis, facilitating the decision for surgical intervention.

Histologically, adrenal hemangiomas are classified as cavernous or capillary types, with the cavernous subtype being more common. Our case confirmed the presence of a cavernous hemangioma, characterized by large blood-filled spaces lined by endothelial cells. Immunohistochemistry typically reveals positivity for CD31, CD34, and *ERG* (erythroblast transformation-specific related gene), consistent with the vascular origin of the tumor [1, 7, 8].

Due to the rarity of adrenal hemangioma, no definitive treatment guidelines exist. Small, asymptomatic, and benign-looking masses can be managed conservatively. However, surgical excision remains the definitive treatment for adrenal hemangiomas, particularly for symptomatic or large lesions [7, 10]. While benign, adrenal hemangiomas are vascular tumors, and complications such as rupture, retroperitoneal bleeding, hypovolemia, anemia, and even acute thrombosis can occur [13]. Laparoscopic adrenalectomy is preferred due to its minimally invasive approach and favorable postoperative outcomes, although open surgery is sometimes necessary when malignancy cannot be definitively ruled out preoperatively [12]. In our case, the patient underwent successful laparoscopic adrenalectomy with no postoperative complications. The long-term prognosis for adrenal hemangioma is excellent, with no recurrence reported in most cases.

The polycythemia workup in our patient was unremarkable, and the condition resolved following phlebotomy. A potential explanation for the transient polycythemia could be a prior COVID-19 infection, which has been associated with polycythemia [14]. One proposed mechanism involves the overactivation of CD169 macrophages, which stimulate the bone marrow to produce erythrocytes [15, 16].

Adrenal hemangiomas are rare benign tumors that are often discovered incidentally during imaging. While typically asymptomatic, their resemblance to more aggressive adrenal tumors can pose diagnostic challenges. Proper recognition of imaging features is essential for accurate diagnosis and appropriate management. This case contributes valuable insights into the understanding of these uncommon tumors. Further research is necessary to elucidate their clinical course and establish optimal management strategies.

## Learning Points

- Adrenal hemangiomas are often detected incidentally during imaging for unrelated conditions.
- Differentiating adrenal hemangiomas from aggressive adrenal tumors requires imaging, biochemical evaluation, and clinical judgment.
- Typical imaging findings include T2 hyperintensity (lightbulb sign) and peripheral enhancement on contrast-enhancement MRI scan.
- Progressive enlargement or indeterminate features necessitate surgical excision, with laparoscopic adrenalectomy offering excellent outcomes and low recurrence rates.

## Data Availability

Original data generated and analyzed during this study are included in this published article.

## Abbreviations

CECT: contrast-enhanced computed tomography
CT: computed tomography
FDG: fluorodeoxyglucose
HU: Hounsfield unit
MRI: magnetic resonance imaging
PET: positron emission tomography
